# Naming CRISPR alleles: endonuclease-mediated mutation nomenclature across species

**DOI:** 10.1007/s00335-017-9698-3

**Published:** 2017-06-06

**Authors:** Michelle N. Knowlton, Cynthia L. Smith

**Affiliations:** Mouse Genome Informatics, The Jackson Laboratory, Bar Harbor, 04609 USA

## Abstract

The widespread use of CRISPR/Cas and other targeted endonuclease technologies in many species has led to an explosion in the generation of new mutations and alleles. The ability to generate many different mutations from the same target sequence either by homology-directed repair with a donor sequence or non-homologous end joining-induced insertions and deletions necessitates a means for representing these mutations in literature and databases. Standardized nomenclature can be used to generate unambiguous, concise, and specific symbols to represent mutations and alleles. The research communities of a variety of species using CRISPR/Cas and other endonuclease-mediated mutation technologies have developed different approaches to naming and identifying such alleles and mutations. While some organism-specific research communities have developed allele nomenclature that incorporates the method of generation within the official allele or mutant symbol, others use metadata tags that include method of generation or mutagen. Organism-specific research community databases together with organism-specific nomenclature committees are leading the way in providing standardized nomenclature and metadata to facilitate the integration of data from alleles and mutations generated using CRISPR/Cas and other targeted endonucleases.

## Introduction

The emergence of targeted endonucleases as a means for somatic and germline modification provides a versatile approach to mutagenesis and genetic engineering across species. Transcriptional activator-like effector nucleases (TALENs), zinc finger nucleases (ZFN), and clustered regularly interspaced short palindromic repeats with Cas9 nucleases (CRISPR/Cas9) have been successful in more than forty organisms, with uses spanning the creation of human disease models, essential basic research and commercial modifications to agricultural organisms (Table 1; reviewed in Harrison et al. 2014 and; Carroll 2014).

**Table 1:** Targeted endonucleases (CRISPR, TALEN, ZFN) have been used in many species to generate somatic and germline modifications in organisms

Organism	Endonuclease targeting system	Reference
Vertebrates		
Axolotl	CRISPR, TALEN	Fei et al. (2014), Flowers et al. (2014)
Catfish	CRISPR, TALEN, ZFN	Dong et al. (2011), Dong et al. (2014), Li M et al. (2016)
Cattle	CRISPR, TALEN, ZFN	Wu et al. (2015), reviewed in Tang et al. (2015)
Frog	CRISPR, TALEN, ZFN	Reviewed in Shi et al. (2017)
Goat	CRISPR, TALEN	Reviewed in Menchaca et al. (2016)
Medaka	CRISPR, TALEN, ZFN	Ansai and Kinoshita (2014), Guan et al. (2014), Wang and Hong (2014)
Mouse	CRISPR, TALEN, ZFN	Reviewed in Sander and Joung (2014)
Monkey	CRISPR, TALEN	Niu et al. (2014), Wan et al. (2015)
Newt	TALEN	Hayashi T et al. (2014)
Pig	CRISPR, TALEN, ZFN	Sato et al. (2014), reviewed in Tang et al. (2015)
Rabbit	CRISPR, TALEN, ZFN	Flisikowska et al. (2011), Honda et al. (2015), Wang et al. (2014b)
Rat	CRISPR, TALEN, ZFN	Reviewed in Mashimo T (2014)
Rainbow trout	ZFN	Yano et al. (2012)
Sheep	CRISPR, TALEN, ZFN	Reviewed in Menchaca et al. (2016), Zhang et al. (2016a, b, c)
Tilapia	CRISPR, TALEN	Li et al. (2013a, b), Li et al. (2014)
Zebrafish	CRISPR, TALEN, ZFN	Reviewed in Auer et al. (2014)
Invertebrates		
Butterfly	CRISPR, TALEN, ZFN	Market et al. (2016), Merlin et al. (2013)
Cricket	CRISPR, TALEN, ZFN	Awata et al. (2015), Watanabe (2012)
Freshwater flea	CRISPR, TALEN	Naitou et al. (2015), Nakanishi et al. (2014), Nakanishi et al. (2015)
Fruit fly	CRISPR, TALEN, ZFN	Reviewed in Lin et al. (2014)
Mosquito	CRISPR, TALEN, ZFN	Aryan et al. (2013), review in Reegan et al. (2017)
Plasmodium	CRISPR, TALEN, ZFN	Smidler at al. (2013), Straimer et al. (2012), reviewed in Singer and Frischknecht (2017)
Roundworm	CRISPR, TALEN, ZFN	reviewed in Gaj et al. (2013), Waaijers and Boxem (2014)
Sea squirt	CRISPR, TALEN, ZFN	Kawai et al. (2012), Sasaki et al. (2014), Treen et al. (2014)
Silkworm	CRISPR, TALEN, ZFN	Reviewed in Xu and O’Brochta (2015)
Plants		
Barley	CRISPR, TALEN	Lawrenson et al. (2015), Wendt et al. (2013)
Bunchgrass	TALEN	Shang et al. (2013a)
Cabbage	CRISPR, TALEN	Lawrenson et al. (2015), Sun et al. (2013)
Corn	CRISPR, TALEN, ZFN	Char et al. (2015), Shukla et al. (2009), Somaratne et al. (2017)
Chlamydomonas	CRISPR, ZFN	Shin et al. (2016), Sizova et al. (2013)
Duncan grapefruit	CRISPR	Jia et al. (2016)
Liverwort	CRISPR	Sugano et al. (2014)
Petunia	CRISPR, ZFN	Marton et al. (2010), Zhang B et al. (2016a, b, c)
Rice	CRISPR, TALEN	Li et al. (2012), reviewed in Belhaj et al. (2013)
Sorghum	CRISPR	Jiang et al. (2013)
Soybean	CRISPR, TALEN, ZFN	Curtin et al. (2011), Haun et al. (2014), Jacobs et al. (2015)
Sugarcane	TALEN	Jung and Altpeter (2016)
Sweet orange	CRISPR	Jia and Wang (2014)
Thale cress	CRISPR, ZFN	Li et al. (2013a, b), Zhang F et al. (2010), Zhang Z et al. (2016 **)**
Tobacco	CRISPR, TALEN, ZFN	Li et al. (2013a, b), Townsend et al. (2009), Zhang et al. (2013)
Wheat	CRISPR, TALEN	Shan et al. (2013b), Wang et al. (2014a)

Adapted from Harrison et al. 2014 and Carroll 2014. Due to the rapid pace development of targeted endonuclease-mediated mutation technologies, this table should be considered representative but not all-inclusive

**Fig. 1:**
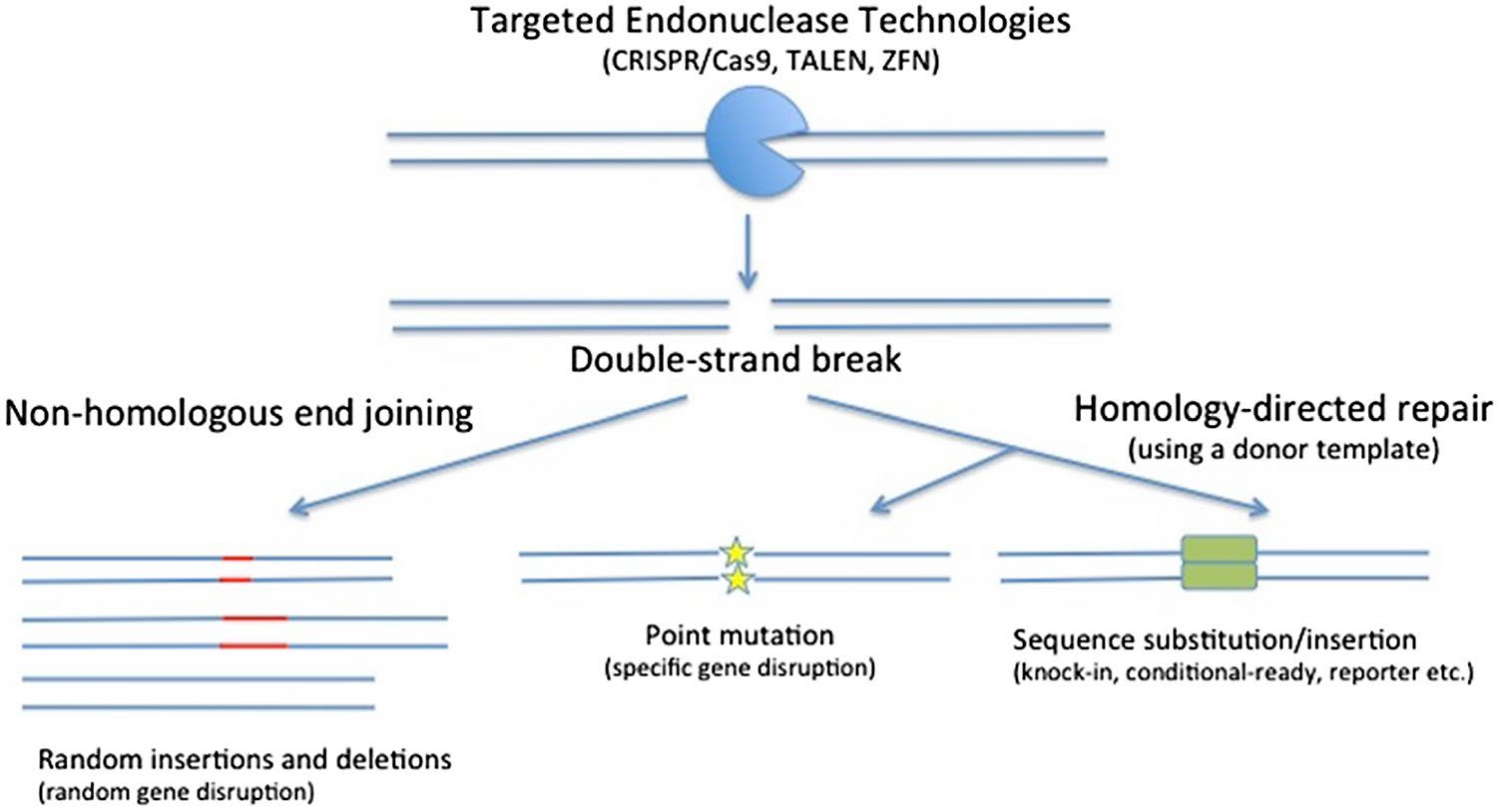
Targeted endonucleases technologies such as CRISPR/Cas9, TALEN, and ZFN induce targeted double-strand breaks that can be repaired via (1) error prone, non-homologous end joining to produce insertions and deletions, or (2) homology-directed repair with a donor template to produce specific point mutations or a variety of knock-ins (conditional ready, reporter etc.)

The use of CRISPR/Cas9 technology is quickly surpassing other methods for rapid generation of an array of single gene and multiplex mutations with minimal sequence constraints often associated with ZFN and TALENs (reviewed in Seruggia and Montoliu 2014). All three technologies induce targeted double-strand breaks that are repaired through either error-prone, non-homologous end joining (NHEJ) or homology-directed recombination (HDR) with a donor template depending on experimental conditions to produce a variety of mutations ranging from insertions and deletions to sequence replacement **(**Fig. 1; reviewed in Carroll 2014; Chen et al. 2014; Guha et al. 2017; Harrison et al. 2014 **)**. Thus, a single targeting sequence can produce an array of alleles from those containing a single-nucleotide deletion or insertion to alleles with large gene deletion (65 kb) or cassette insertion up to 5 kb (Zhang et al. 2015). The ability to generate so many mutations from a single targeting sequence makes CRISPR/Cas9 a powerful tool for reverse genetics, targeted allele generation, and reversion of existing mutation (reviewed in Guha et al. 2017). In addition, the number and diversity of mutations and alleles created presents a challenge to unambiguous identification, integration of data within organism-specific databases, and cross-species data mining using external resources available at InterMine and the fledgling Alliance of Genome Resources (see “Summary”).

## Nomenclature for a wide variety of endonuclease-mediated mutations

Unlike traditional gene targeting methods, endonuclease-mediated mutation can produce a number of outcomes depending on the availability of a repair template, cell cycle and the nuclease variant used. Single-nucleotide deletions, insertions and substitutions, specific sequence replacement or insertion, large deletions or genomic rearrangements (e.g., inversions or translocations), up-regulation of specific endogenous genes, altered histone modifications or DNA methylation, or insertion of fluorescent proteins have all been achieved by these methods (reviewed in Guha et al. 2017, reviewed in; Sander and Joung 2014; Wolfs et al. 2016; Zhang et al. 2017). In addition to biasing repair toward NHEJ, HDR or modification of histones, CRISPR/Cas technology can be used to target more than one gene at a time (reviewed in Rocha-Martins et al. 2015).

With the prospects of creating numerous mutations per experiment, standardized nomenclature is critical to correctly identify individual heritable alleles. To be useful, symbols must not only be unambiguous but also specific, concise, and informative. While gene nomenclature has been standardized in many species, allele or mutant nomenclature guidelines vary. The major organism databases (Table 2) integrate genetic information and act as authoritative sources for gene, allele, and/or strain nomenclature. Although no standardized nomenclature exists across species to designate endonuclease-mediated mutations, the organism databases have taken two general approaches to CRISPR allele nomenclature: (1) specific endonuclease-mediated mutation nomenclature, (2) standard allele and mutation nomenclature, or (3) adopting HGNC nomenclature guidelines.

**Table 2:** Summary of organism-specific nomenclature resources

Organism	Resource (website)
*Arabidopsis thaliana*	TAIR: The Arabidopsis Information Resource (http://www.arabidopsis.org)
*Caenorhabditis elegans*	WormBase (http://www.wormbase.org)
Cattle	The Bovine Genome Database (http://www.bovinegenome.org)
Chicken	BirdBase (http://www.birdbase.arizona.edu/birdbase)
Drosophila	FlyBase (http://www.flybase.org)
Frog	Xenbase (http://www.xenbase.org)
Maize	MaizeGDB: Maize Genetics and Genomics Database (http://www.maizegdb.org)
Mice	MGI: Mouse Genome Informatics (http://www.informatics.jax.org)
Pig	Pig Genome Informatics System (http://www.pig.genomics.org.cn)
Rat	RGD: Rat Genome Database (http://www.rgd.mcw.edu)
Yeast	SDG: Saccharomyces Genome Database (http://www.yeastgenome.org)
Zebrafish	ZFIN: Zebrafish Information Network (http://www.zfin.org)

## Unique endonuclease-mediated mutation allele nomenclature (mouse, rat, and *Xenopus*)

The International Committee on Standardized Genetic Nomenclature for Mice and the Rat Genome Nomenclature Committee has established guidelines for the nomenclature of genes, genetic markers, alleles, and mutations in mouse (http://www.informatics.jax.org; Blake et al. 2017) and rat (http://rgd.mcw.edu; Shimoyama et al. 2015). Allele symbols are composed of the modified gene with the allele information in superscript (http://www.informatics.jax.org/mgihome/nomen/gene.shtml#endim). The allele information includes method of generation, serial number for the number of alleles generated by the laboratory in the gene, and laboratory code registered with ILAR (International Laboratory Animal Research; http://dels.nas.edu/global/ilar/lab-codes). For example, *Apoc2* ^*em1Arem*^ represents the first endonuclease-mediated mutation generated in the apolipoprotein C-II gene by the laboratory of Dr. Alan Remaley (Sakurai et al. 2016). Likewise, *Mecp2* ^*em1Sage*^ represents the first endonuclease-mediated mutation generated in the methyl CpG binding protein 2 gene by Sigma Advanced Genetic (Wu et al. 2016). The endonuclease-mediated mutation (em) includes mutations generated by TALENs, ZFNs, CRISPR/Cas, and any potential future technologies that utilize targeted endonucleases to nick the DNA and modify the genome.

Beyond standard em allele nomenclature, MGI utilizes chromosomal aberration nomenclature when CRISPR-guided target sequences flanking more than one gene (http://www.informatics.jax.org/mgihome/nomen/anomalies.shtml). For example, Del(5Kit-Nmu)2Staka represents an endonuclease-mediated mutation that results in a deletion on chromosome five spanning from KIT proto-oncogene receptor tyrosine kinase to neuromedin U that is the second deletion generated in the laboratory of Dr. Satoru Takahashi (Mizuno et al. 2015). The ability to associate alleles to more than one gene via a mutation involves relationship provides access to this allele via all the markers affected in the deletion (Eppig et al. 2015).

To facilitate searching for em alleles whether the allele targets one or more genes, MGI tags the alleles with generation-type ‘endonuclease-mediated’ and the exact endonuclease technology is further specified in the molecular details and origin in MGI and RGD, respectively, on allele detail pages. For example, *Smg9* ^*em1J*^ (http://www.informatics.jax.org/allele/key/853643; Shaheen et al. 2016) is annotated with the allele generation attribute ‘endonuclease-mediated mutation.’ The mutation details describe the allele in detail. The combination of endonuclease-mediated mutation-specific allele symbols and metadata tags identifies CRISPR/Cas-generated alleles and facilitates phenotypic and disease-association analysis in MGI and RGD.

Xenbase (http://www.xenbase.org; James-Zorn et al. 2015 and Karpinka et al. 2015) is the research community resource that integrates data for *Xenopus tropicalis* and *Xenopus laevis*. The frog community utilizes nomenclature based on the nomenclature guidelines established by the International Committee on Standardized Genetic Nomenclature for Mice. Endonuclease-mediated mutations are represented by nomenclature that includes the three letter species code, period, gene symbol, method of generation, a serial number for mutations in the particular locus generated by a particular laboratory and the ILAR registered laboratory code. For example, *Xtr.gsc* ^*em1Cho*^ represents the first endonuclease-mediated mutation in the goosecoid homeobox gene from the laboratory of Dr. Cho in *Xenopus tropicalis* (Blitz et al. 2016).

## Use of existing allele nomenclature for endonuclease-mediated mutations (Arabidopsis, *C. elegans*, Drosophila, Maize, Yeast, and Zebrafish)

A number of model organisms and research organism databases utilize existing organism-specific standardized nomenclature to describe CRISPR/Cas-generated alleles and mutants.

The Arabidopsis Information Resource (TAIR; https://www.arabidopsis.org/; Berardini et al. 2015) is the authoritative source for *Arabidopsis thaliana* gene and mutant nomenclature. Alleles are designated by the gene symbol, hyphen, and serial number for mutations in that gene (The Arabidopsis Information Resource 2017). For example, *idm3-4* represents the fourth mutation in the increased DNA methylation 3 gene (Lang et al. 2015). The mutagen field on the allele detail page reflects generation using CRISPR technology.

WormBase (http://www.wormbase.org; Harris et al. 2010), a community research resource for *Caenorhabditis elegans* and related nematodes, alleles are described by a one- or two-letter laboratory code which refers to the laboratory of isolation, registered at the Caenorhabditis Genetic Center (CGC; https://cbs.umn.edu/cgc/home) and serial number corresponding to the number of mutations generated by the specific laboratory. For example, *ect2*(*xs110*) is the one hundred tenth variant generated by the laboratory of Dr. M Glotzer in the ECT2 (mammalian Rho GEF) homolog gene (Zhang and Glotzer 2015). While optional suffixes exist for generation methods (e.g., transposon-excision, te) and consequence (e.g., temperature-sensitive, ts), there is no current suffix for endonuclease-mediated mutation. A method of generation tag specifies generation by CRISPR technology and can be viewed using the Tree Display tool on the variation detail page. The WormBase Query Language can be used to search for “CRISPR/Cas9” as a production method. Additionally, WormMine release WS259 will allow users to search for engineered alleles (personal communication from WormBase).

Flybase (http://flybase.org/; Gramates et al. 2017) is a database of Drosophila genes and genomes. Allele nomenclature consists of the species symbol, backslash, gene, and allele name provided by the generating laboratory in superscript (Flybase 2017). For example, *Dmel*\*e* ^*HDR*−*CRISPR*^ is the HDR-CRISPR mutation in the *Drosophila melanogaster* ebony gene. There is no nomenclature requirement to include CRISPR as a part of the allele symbol. The mutagen field on the allele detail page labels this allele as being generated by CRISPR/Cas technology.

MaizeGDB (http://www.maizegdb.org; Andorf et al. 2016) is a maize genetics and genomics database. Allele nomenclature is composed of the gene symbol plus a letter or numbers corresponding to the mutation. The generating laboratory assigns the numbers and or line name. The number corresponds to the serial number of all mutations in the given gene. For example, *ms10-CRISPR1* represents the CRISPR1 mutation in the male sterile 10 gene (Somaratne et al. 2017). There is no requirement to include CRISPR in the allele symbol as a part of the laboratory line number or name. The mutagen field on the variation record captures the method of generation by CRISPR/Cas-technology.

Saccharomyces Genome Database (http://www.yeastgenome.org/; Cherry et al. 2012) is a research database dedicated to the budding yeast *Saccharomyces cerevisiae*. They are the authoritative source for yeast gene and mutant nomenclature (Cherry 1995). Mutant yeast nomenclature utilizes characters that represent the nature of the genomic modification in addition to gene symbols. For example, *ade6::URA4* would represent a disruption in the phosphoribosylformylglycinamidine synthase gene with the insertion of a functional dihydroorotase gene. There is no current symbol to represent endonuclease-mediated mutations such as those generated by CRISPR/Cas technology (personal communication SGD Project 2017).

In the Zebrafish Information Network (https://zfin.org/; Howe et al. 2013), allele and mutation symbols are based on the resulting genetic disruption produced by a given laboratory using laboratory codes registered with ZFIN. For example, *kif5ba* ^*ae11*^ represents the eleventh mutation in the kinesin family member 5B gene generated by the laboratory of Dr. Marlow (Campbell et al. 2015). While the nomenclature schema does not include a character to represent CRISPR-generated alleles, information about the method of generation is stated as an experiment-specific mutagen in the protocol field of the allele detail page and mutagen field on the gene detail page, respectively.

## Standard HGNC allele nomenclature (cattle, chicken, and pig)

While databases exist for genomic research in several agricultural organisms (see Table 2), their focus is on genome annotation and spontaneous or engineered alleles are not generally annotated for these organisms. In the absence of standard allele nomenclature, several agricultural organisms have adopted guidelines in keeping with HUGO Gene Nomenclature Committee (HGNC) guidelines (http://www.genenames.org/, Yates et al. 2017). The general recommendation by HGNC is to represent an allele using the gene symbol, an asterisk and the allele symbol on the same line (e.g., PGM1*1; Wain et al. 2002). This includes livestock species such as cattle, chicken, and pig (Burt et al. 2009; Hu et al. 2011, 2014).

## Summary

The continued development of new technologies for generating germline modifications in different species poses a nomenclature challenge for the scientific community. While nomenclature conventions continue to evolve when the need arises to represent new information, the stability and concise nature of unambiguous and specific symbols and names is the purpose of standardized nomenclature. Although the various organism-specific communities have opted for different approaches, they nevertheless provide researchers with the ability to identify endonuclease-mediated mutations by symbol and/or metadata tag.

In addition to promoting the reproducibility of data by unambiguously identifying alleles, standard nomenclature facilitates the integration and analysis of phenotypic data across species. Tools are emerging to allow for high-level comparison of annotated data across species at the level of the gene or phenotype. InterMine offers a platform for cross species comparison (http://intermine.org/; Lyne et al. 2015). Mines have been established for several model organisms, including fly, frog, human, mouse, rat, worm, yeast and zebrafish, and for specialized research area such as mitochondrial proteomics and modENCODE data (see http://intermine.org/). The Alliance of Genome Resources (AGR; http://home.alliancegenome.org), an intersection between six major model organism databases (MGI, RGD, SGD, ZFIN, FlyBase and WormBase), aims to allow for cross species comparison for analysis of genetic, phenotypic, and disease-related annotations. The Monarch Initiative (monarchinitiative.org; Mungall et al. 2017) integrates genotype to phenotype data across multiple species to support biomedical research. MouseNet2 (http://www.inetbio.org/mousenet/; Kim et al. 2016) integrates gene network data, particularly from microarray experiments, for the discovery of novel disease genes and disease pathways.

The rapidly evolving technologies utilizing targeted endonuclease-mediated mutation to create new alleles will continue to generate numerous somatic and germline mutations. These tools will expand our understanding of basic biology and disease models in addition to offering a method to repair or treat existing mutations faster and with greater ease than previous mutagenesis technologies. As scientists continue to refine their ability to induce and repair genetic mutations, the need for unambiguous identification of their tools and genetic products becomes all the more important.
